# Lower extremity amputations in Switzerland: Trends in patients with and without diabetes, 2013–2023

**DOI:** 10.1016/j.ijcrp.2026.200696

**Published:** 2026-08-12

**Authors:** Alexander Schuster, Simon Wolf, Julia Neuenschwander, Madlaina Schöni, Felix W.A. Waibel, Christina Sydler, Lea Attias Widmer, Faranak Rostami, Marc Righini, Nils Kucher, Stefano Barco, Davide Voci

**Affiliations:** a Department of Internal Medicine, Kliniken an der Paar, Aichach/Friedberg, Germany; b Department of Vascular Medicine, University Hospital Zurich, University of Zurich, Zurich, Switzerland; c Department of Vascular Medicine, Kantonsspital Baden, Baden, Switzerland; d Balgrist University Hospital, Department of Technical and Neuro- Orthopaedics, Zurich, Switzerland; e Vascular Center, Spitalzentrum Biel, Biel, Switzerland; f Division of Angiology and Hemostasis, Geneva University Hospitals and Faculty of Medicine, Geneva, Switzerland

**Keywords:** Lower extremity amputation, Diabetes mellitus, Peripheral arterial disease, Epidemiology

## Abstract

**Background and aims:**

Lower extremity amputation (LEA) is a critical public health issue predominantly caused by diabetes mellitus (DM) and peripheral arterial disease (PAD). Switzerland faces a growing diabetes burden amid suboptimal glycemic control. The aim was to analyze LEA trends in Switzerland (2013-2023), comparing diabetic and non-diabetic populations to inform targeted prevention and management strategies.

**Methods:**

A retrospective, nationwide analysis of Swiss hospital discharge data from 2013 to 2023 was conducted. LEA cases were identified via Swiss surgical procedure codes and stratified by diabetes status using ICD-10-GM diagnosis codes. Age-standardized incidence rates and amputation proportions were computed, with Poisson regression models to evaluate risk factors. Outcomes including in-hospital mortality and re-amputation rates were assessed.

From 2013 to 2023, 30,047 lower extremity amputations were performed in Switzerland, with 60% occurring in patients with diabetes and 85% being minor amputations. Crude annual numbers increased, but age-standardized rates remained stable. Patients with diabetes had a 56% higher adjusted risk than those without. Amputation proportions in diabetes-related hospitalizations decreased over time, driven by a reduction in minor amputations, while major amputations remained stable. In-hospital mortality was 4.3%, lowest in type 1 diabetes, and 13% experienced re-amputation within 36 months.

**Conclusions:**

Lower-extremity amputation remains a preventable complication; improved diabetes control and access to early, multidisciplinary foot care are critical to further reduce avoidable limb loss in Switzerland.

## 1 Introduction

Lower extremity amputation (LEA) is a major public health concern, with diabetes mellitus (DM) and peripheral arterial disease (PAD) as the leading causes of non-traumatic limb loss [[1], [2], [3]]. The burden of DM continues to rise, with over 550 million affected individuals globally and an estimated number of 700 million by 2045 [4]. Complications of diabetic foot are a key driver of hospital admissions and amputations. Of the approximately 1.6 million non-traumatic LEAs performed worldwide each year, current data suggest that diabetes is implicated in 61-92% of major amputations and 79-89% of minor amputations [3,5].

In Switzerland, the prevalence of diabetes ranges from 6.2% to 8.7% and glycemic control remains suboptimal with only half of diagnosed patients receiving treatment [6].

Recent studies and multinational analyses across Europe and Northern America have documented a decline in major LEA among people with diabetes, attributed to advances in preventive care, vascular interventions, and multidisciplinary management, whereas minor amputation rates tended to increase or remain stable [[7], [8], [9], [10], [11]].

The overall burden of LEA remains substantial, with persistent high mortality and a shifting demographic profile, including younger age at amputation and increasing comorbidity burden [2,12]. In Switzerland and neighboring regions, single-center studies have highlighted high mortality rates (16–23% at one year), increasing comorbidity frequency, and notable sex differences, with women undergoing major amputations at older ages than men [2]. Social and structural determinants, such as access to care and socioeconomic disparities, continue to drive inequalities in amputation risk [3,13]. The COVID-19 pandemic has further disrupted care pathways, contributing to a post-pandemic surge in LEA rates among patients with diabetes [14].

In Switzerland, there is limited data on LEA trends from the past decade, particularly regarding the differential impact of diabetes. The increasing prevalence of diabetes, suboptimal treatment and glycemic control, and persistent disparities underscore the need for updated epidemiological data to inform targeted prevention strategies and optimize limb salvage efforts in high-risk populations [6]. The aim of the present study was to elucidate time trends in LEA in Switzerland, between 2013 and 2023 with specific focus on differences according to diabetes status. Beyond amputation rates and incidence rate ratios, we additionally characterised the comparator group of patients without diabetes and examined the principal in-hospital outcomes (case fatality and length of stay), post-discharge destination, and the risk of subsequent re-amputation, so as to provide a comprehensive picture of the contemporary burden of LEA in Switzerland and the way it differs by diabetes status.

## 2 Patients and methods

### 2.1 Data source and definitions

We performed a retrospective, nationwide analysis of the Swiss medical statistics, an administrative data collection, covering the years 2013 to 2023. By law, all public and private healthcare institutions are annually required to report all discharge data to the Swiss Federal Statistical Office.

Clinical diagnoses were coded according to the International Classification of Diseases, 10th Revision, German Modification (ICD-10-GM). Surgical procedures, including amputations, were identified through the Swiss Classification of Surgical Operations (CHOP) codes. Hospitals are responsible for ensuring coding accuracy by reconciling recorded diagnoses and procedures with clinical documentation from each hospital stay. At the federal level, data undergo systematic quality control, with annual coding validation completed by the Swiss Federal Statistical Office within two years of the reporting period.

LEA–related hospitalizations were identified through CHOP procedure codes for amputations, which also allowed classification into major (above the ankle joint) and minor (through or below the ankle joint) procedures as proposed in the literature [15]. In addition, ICD-10-GM diagnosis codes were used to determine diabetes status (E10–E11). If a diabetes code was documented at any position of the diagnosis list, the hospitalisation was categorized as diabetes-related; otherwise, it was considered non-diabetes-related. The non-diabetes comparator group does not represent a disease-free population but comprises patients undergoing non-traumatic, non-tumour LEA predominantly in the context of peripheral arterial disease and chronic limb-threatening ischaemia, together with other conditions such as soft-tissue or bone infection, chronic venous disease and end-stage renal disease; it therefore serves as a contemporary reference group against which the additional, diabetes-specific burden of amputation can be quantified. Cases with mention of traumatic amputations (ICD-10-GM: S88, S98, T05, T13, T14.7) or tumor-related amputations (ICD-10-GM: C40–C41 for malignant bone tumors; C49 for malignant soft tissue tumors) were excluded, to focus on non-traumatic, disease-related events. ICD-10-GM codes for concomitant diseases and CHOP codes for performed procedures are presented in Supplementary Table 1–2*.*

This study was conducted in accordance with the Reporting of studies Conducted using Observational Routinely-collected Data (RECORD) standards [16]. As the database provides fully anonymized information, written informed consent by patients and protocol approval by the institutional ethics committee and ethical approval are not necessary.

### 2.2 Statistical analysis

We calculated crude and age-standardized incidence rates for all in-hospital amputations as the number of cases per 100,000 general population per year and the proportion of hospital admissions per 1000 hospital admissions. Patients were stratified based on the presence of DM and type of DM. Direct age-standardization was performed using the 2013 European standard population. The in-hospital case fatality rate was calculated as the number of in-hospital deaths per 100 amputation related hospitalizations. To study longitudinal time trends, we utilized Poisson regression models with year as a categorical variable. To study re-hospitalisation, we employed the Kaplan-Meier method, patients were stratified according to the location of the first identified amputation. The Swiss medical statistics contain an anonymized patient linkage identifier that allows separate hospitalisations of the same individual to be connected across admissions, which underlies the re-amputation analysis. For the re-amputation analysis, the first recorded amputation served as the index event and was classified as major or minor, and subsequent amputations were identified as later admissions carrying an amputation procedure code. Two units of analysis were therefore applied. All descriptive analyses, amputation rates, amputation proportions and Poisson regression models were performed at the level of the amputation-related hospitalisation and included both first and repeat amputations, so that a patient undergoing more than one amputation during the study period contributed more than one observation (30,047 amputations in 26,117 individual patients). The re-amputation analysis, by contrast, was performed at the patient level: each patient contributed a single index amputation, and any subsequent amputation was counted as an outcome event rather than as a new index event. Unless stated otherwise in the corresponding table or figure legend, all reported results include both first and repeat amputations.

Data is presented either as number and percent or, for continuous variables, as median and interquartile range. All rates and proportions are presented as crude and age-standardized nationwide rates. Rates and frequencies are presented with appropriate 95% confidence intervals (CI). Trends in figures were visualized using locally estimated scatter point smoothing. The statistical analysis was performed using R version 4.5.0.

## 3 Results

### 3.1 Study population

From 2013 to 2023, a mean mid-year population of 8.5 million lived in Switzerland with an increasing trend over time. A mean 1.4 million hospitalizations were recorded per year. A total of 30,047 LEA were recorded in 26,117 individual patients. Of these amputations, 11,951 (40%) had no DM, 923 (3%) had type 1 DM, and 17,173 (57%) had type 2 DM. Most of the patients were men, amounting to 70% of total. The median age was 75 years (Q1-Q3: 66–82), with the highest age observed in patients without diabetes (median 77 years, Q1-Q3: 67–85) vs those with diabetes type 1 (median 61 years, Q1-Q3: 53–70) and those with diabetes type 2 (median 74 years, Q1-Q3: 66–81). The most prevalent comorbidity was peripheral arterial disease, present in 64% of all cases of LEA and most prevalent in patients with type 2 DM (72%). Comorbidities such as chronic kidney disease and arterial hypertension were also frequent, particularly among patients with DM (Table 1). Minor amputations accounted for approximately 85% of all procedures. Unless stated otherwise, all analyses reported below include both first and repeat amputations.

**Table 1:** Baseline characteristics and comorbidities.

Characteristic	Overall, n = 30,047	No diabetes, n = 11,951	DM1, n = 923	DM2, n = 17,173
Men, n (%)	21,084 (70.2%)	7215 (60.4%)	664 (71.9%)	13,205 (76.9%)
Age (years), median (IQR: Q1-Q3)	75 (66, 82)	77 (67, 85)	61 (53, 70)	74 (66, 81)
**Comorbidities, n (%)**				
Obesity, n (%)	1285 (4.3%)	243 (2.0%)	22 (2.4%)	1020 (5.9%)
Class I	0	0	0	0
Class II	95 (0.3%)	16 (0.1%)	0 (0.0%)	79 (0.5%)
Class III	388 (1.3%)	67 (0.6%)	7 (0.8%)	314 (1.8%)
Peripheral arterial disease	19,465 (64.8%)	6368 (53.3%)	643 (69.7%)	12,454 (72.5%)
Chronic kidney disease	11,028 (36.7%)	3255 (27.2%)	390 (42.3%)	7383 (43.0%)
Chronic venous disease	1190 (4.0%)	517 (4.3%)	15 (1.6%)	658 (3.8%)
Arterial hypertension	20,165 (67.1%)	6704 (56.1%)	619 (67.1%)	12,842 (74.8%)
Coronary artery disease	9568 (31.8%)	2816 (23.6%)	302 (32.7%)	6450 (37.6%)
Heart failure	4044 (13.5%)	1426 (11.9%)	79 (8.6%)	2539 (14.8%)
Alcohol abuse	1352 (4.5%)	712 (6.0%)	21 (2.3%)	619 (3.6%)
Foot ulcer	10,296 (34.3%)	3442 (28.8%)	345 (37.4%)	6509 (37.9%)
Osteomyelitis	13,568 (45.2%)	4441 (37.2%)	537 (58.2%)	8590 (50.0%)
Sensory neuropathy	10,639 (35.4%)	194 (1.6%)	647 (70.1%)	9798 (57.1%)
Previous amputation	4973 (16.6%)	1097 (9.2%)	266 (28.8%)	3610 (21.0%)
Liver disease	900 (3.0%)	376 (3.1%)	20 (2.2%)	504 (2.9%)
Cerebrovascular disease	1448 (4.8%)	521 (4.4%)	43 (4.7%)	884 (5.1%)
Chronic pulmonary disease	2593 (8.6%)	1246 (10.4%)	35 (3.8%)	1312 (7.6%)
Active or history of cancer	1547 (5.1%)	891 (7.5%)	26 (2.8%)	630 (3.7%)

Abbreviations: n: number; DM1: Diabetes Mellitus type 1; DM2: Diabetes Mellitus type 2; IQR: Interquartile Range (Q1-Q3).

*Table 1. All amputations recorded during the study period are included, i.e. both first and repeat amputations; the unit of analysis is the amputation-related hospitalisation.

### 3.2 Trends in amputation rates

Over the 11-year period, the absolute number of amputations (with and without DM) showed a slight increase from 2447 in 2013 to 3143 in 2023, corresponding to an increase in the crude amputation rate (per 10,000 general population) from 3.2 (95% CI: 3.2 - 3.3) in 2013 to 3.5 (95% CI: 3.4 - 3.7) in 2023. The main driver of this increase were minor amputations. The age-standardized amputation rate remained stable over the study period (2.0 [95% CI: 2.0 - 2.0] per 10,000 in 2013 to 2.1 [95% CI: 2.0 – 2.1] per 10,000 in 2023). (Table S3).

**Fig. 1:**
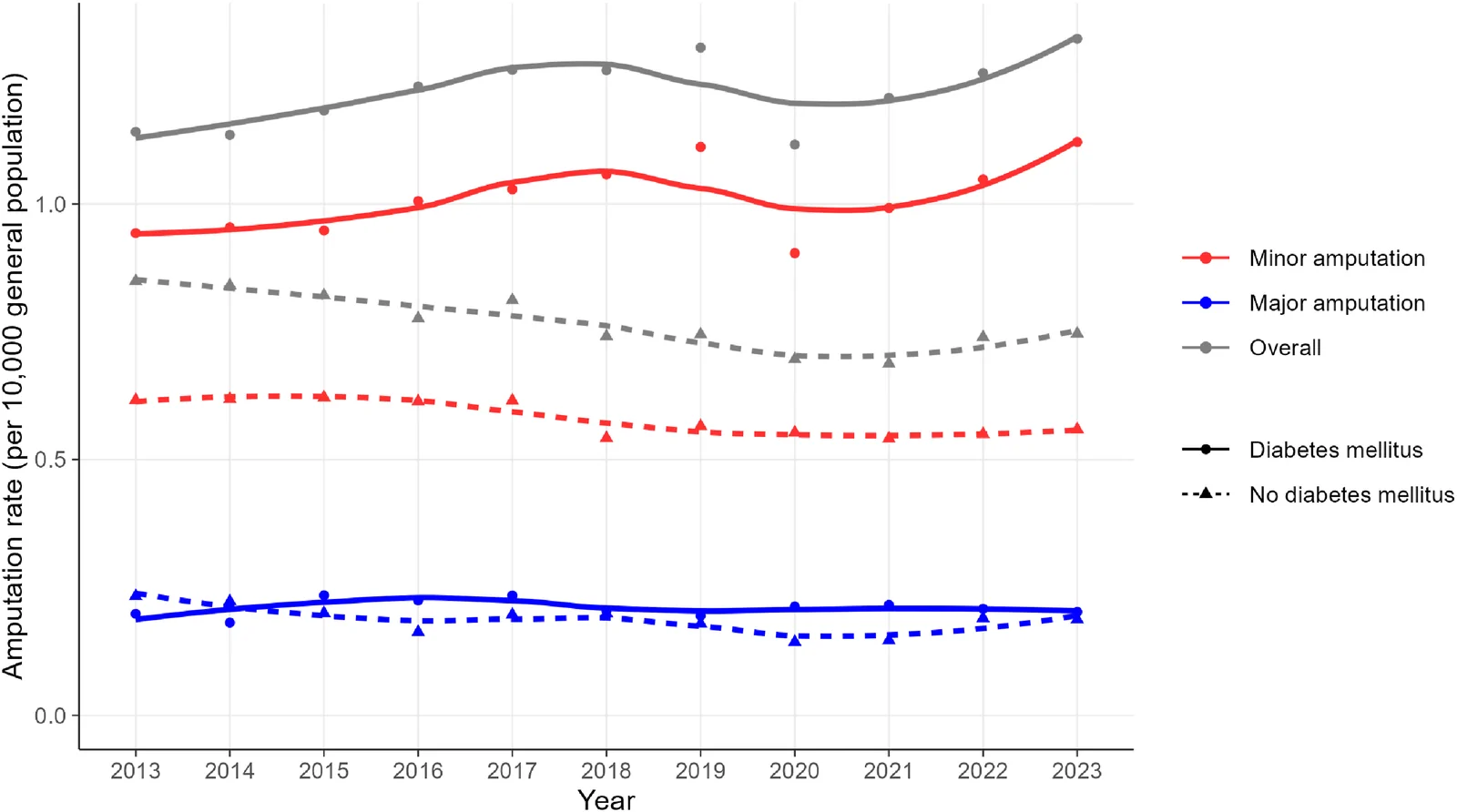
Age-standardized incidence rate (per 10,000 general population) in patients with and without diabetes Fig. 1. Time-series plot showing temporal trends in the age-standardized incidence rate (per 10,000 general population) of lower-extremity amputations among patients with and without diabetes between 2013 and 2023. Separate lines depict minor, major, and overall amputations, stratified by diabetes status. All amputations recorded during the study period are included, i.e. both first and repeat amputations; the unit of analysis is the amputation-related hospitalisation.

Amputation rates were consistently higher in patients with DM (both type 1 and type 2) compared to patients without DM (Table S4, Fig. 1). Over time, age-standardized rates showed a slight increase in total and minor amputations among patients with DM, while rates for major amputations remained stable. In contrast, patients without DM presented with a stable age-standardized amputation rate over time for both minor and major amputations.

**Fig. 2:**
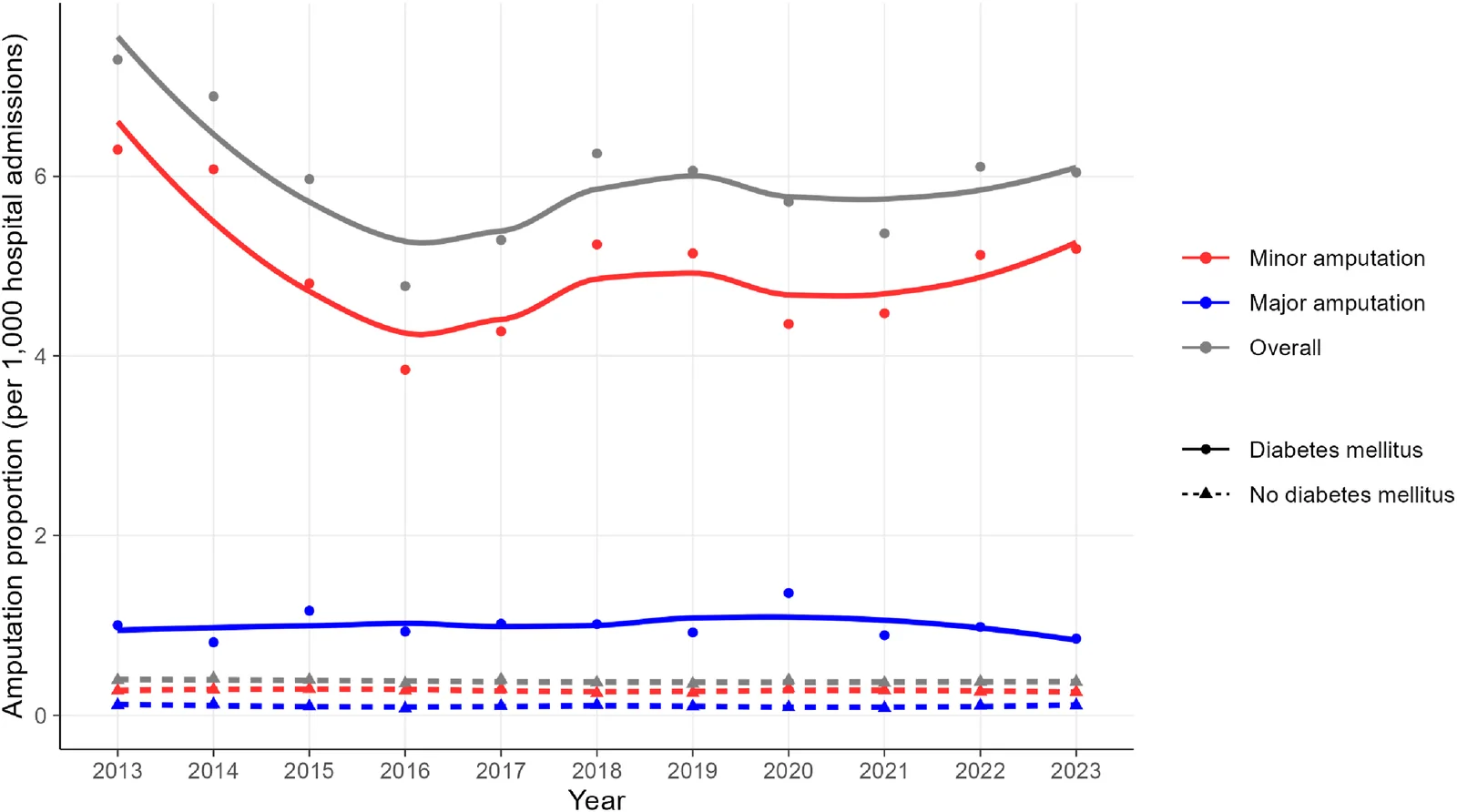
Proportion of hospitalizations (per 1000 hospital admissions) in patients with and without diabetes Fig. 2. Time-series plot graph illustrating trends in the proportion of hospitalizations (per 1000 hospital admissions) of lower-extremity amputations among patients with and without diabetes between 2013 and 2023. Separate lines depict minor, major, and overall amputations, stratified by diabetes status. All amputations recorded during the study period are included, i.e. both first and repeat amputations; the unit of analysis is the amputation-related hospitalisation.

Patients with DM exhibited much higher proportions of amputations during hospitalisation compared with non-diabetic patients (Table S5, Fig. 2). Additionally, over the study period, the age-standardized proportion of amputations among patients with DM-related hospitalisation decreased from 7.3 (95% CI: 6.3 – 34.6) per 1000 diabetes-related hospitalizations in 2013 to 6.0 (95% CI: 5.4 – 24.0) per 1000 in 2023. This decreasing trend was particularly apparent before the COVID-19 pandemic.

The primary driver of this decreasing trend was the decrease in the age-standardized proportion of minor amputations, decreasing from 6.3 (95% CI: 5.3 – 33.8) per 1000 DM-related hospital admissions to 5.2 (95% CI:4.6 – 23.3) per 1000 DM-related hospital admissions. The proportion of major amputation remained stable (1.0 [95% CI: 0.7 – 30.1] 1000 DM-related hospital admissions in 2013 vs. 0.9 [95% CI: 0.6 – 20.2] per 1000 DM-related hospital admissions in 2023). Among DM subtypes (Table S6, Fig. S1 and S2), both crude and age-standardized amputation proportions were higher for patients with type 1 DM than type 2, but only type 2 patients experienced a decrease in the age-standardized AP for minor amputations over time. In patients without DM, the age-standardized amputation proportion remained stable at around 0.4 (95% CI: 0.4 – 0.4) per 1000 hospitalizations for both minor and major amputations.

### 3.3 Incidence rate ratios and predictors

Poisson regression analysis (Table 2 and Table S8) revealed that, after adjustment for age, sex, type of LEA, and calendar year, people with DM had a 56% higher risk of lower extremity amputation compared to those without diabetes (IRR 1.56, 95% CI: 1.53–1.60). Notably, there was a drop in amputation rates during the years 2020 (IRR 0.89, 95% CI: 0.84–0.94) and 2021 (IRR 0.94, 95% CI: 0.89–0.99). Male sex was strongly associated, with men being nearly three times as likely to undergo LEA compared to women (IRR 2.93, 95% CI: 2.86–3.01). Age was the most powerful predictor: individuals aged 75 years or older had almost a 55-fold (IRR 55.4, 95% CI: 50.7–60.7) increased risk of amputation compared to the reference group aged 35–44 years. A similar pattern was observed while analyzing the proportion of hospitalizations associated with LEA (Table 3 and Table S9). Consistent with the analysis of the incidence rate, patients with DM had a markedly higher risk compared to those without, with a relative risk 8.85 times greater (95% CI: 8.64–9.07). Likewise, male sex and advanced age remained strongly associated with amputation risk among hospitalized patients.

**Table 2:** Incidence rate ratio of lower extremity amputation in people with diabetes, compared with those without diabetes, adjusted for age and sex: results of the Poisson models.

Characteristic	IRR	95% CI	p-value
Diabetes mellitus (vs. no diabetes mellitus)	1.56^a^	1.53, 1.60	<0.001
Male sex (vs. female sex)	2.93	2.86, 3.01	<0.001
Age group			
35-44	Reference	Reference	
45-54	3.04	2.75, 3.36	<0.001
55-64	9.75	8.89, 10.7	<0.001
65-74	24.6	22.4, 26.9	<0.001
>75	55.4	50.7, 60.7	<0.001
Major amputation (vs. minor amputation)	0.23	0.23, 0.24	<0.001

Abbreviations: IRR: Incidence Rate Ratio; CI: Confidence Interval.

a Table 2 is calculated with the general population as denominator. All amputations recorded during the study period are included, i.e. both first and repeat amputations; the unit of analysis is the amputation-related hospitalisation.

**Table 3:** Incidence rate ratio of the proportion of hospitalisation of lower extremity amputation in people with diabetes, compared with those without diabetes, adjusted for age and sex: results of the Poisson models.

Characteristic	IRR	95% CI	p-value
Diabetes mellitus (vs. no diabetes mellitus)	8.85^a^	8.64, 9.07	<0.001
Male sex (vs. female sex)	2.02	1.97, 2.07	<0.001
Age group			
35-44	Reference	Reference	
45-54	2.02	1.82, 2.23	<0.001
55-64	3.29	3.00, 3.62	<0.001
65-74	4.20	3.84, 4.61	<0.001
>75	5.14	4.70, 5.64	<0.001
Major amputation (vs. minor amputation)	0.23	0.23, 0.24	<0.001

Abbreviations: IRR: Incidence Rate Ratio; CI: Confidence Interval.

a Table 3 is calculated with the hospital admissions as denominator. All amputations recorded during the study period are included, i.e. both first and repeat amputations; the unit of analysis is the amputation-related hospitalisation.

### 3.4 Outcome and reamputation risk

**Fig. 3:**
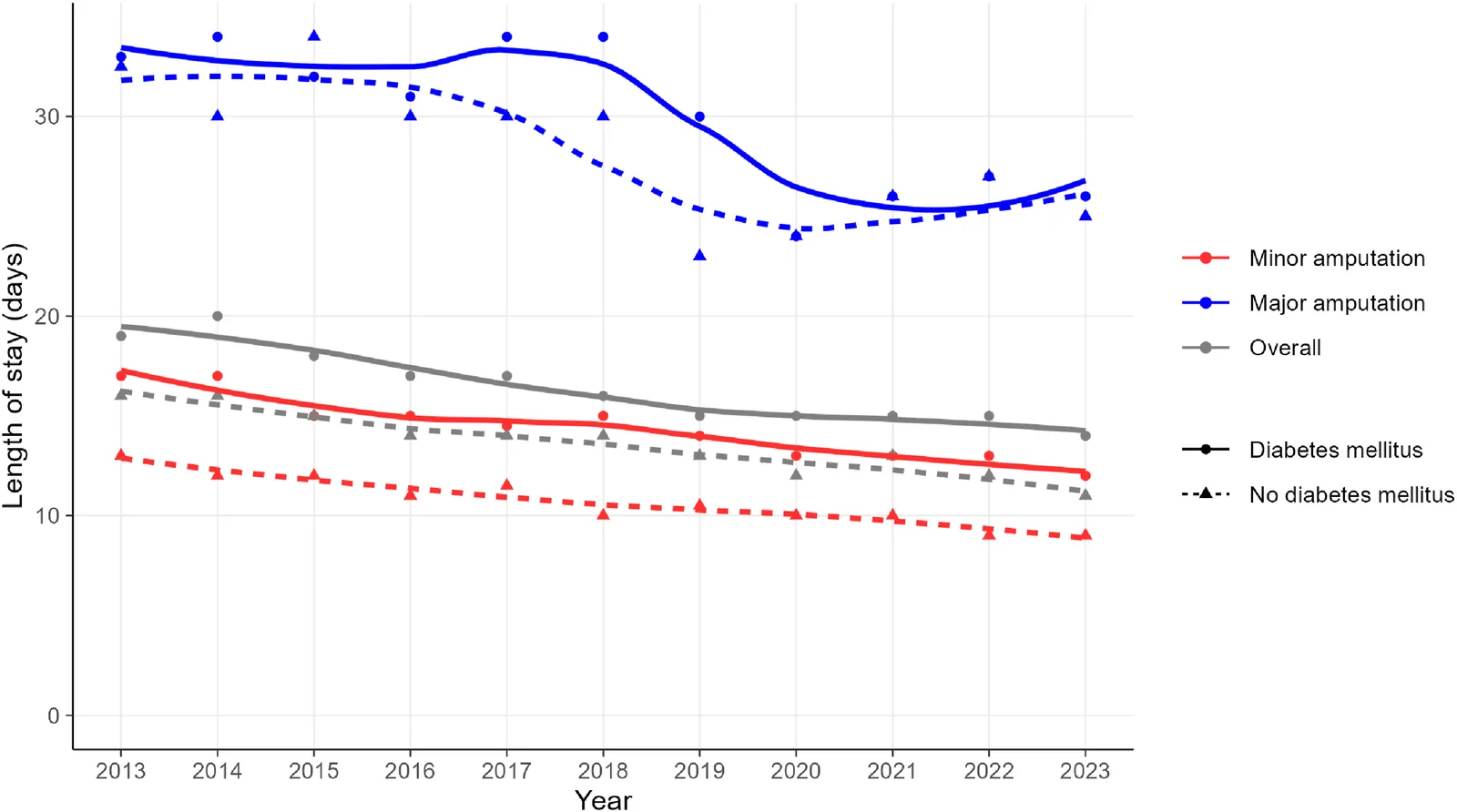
Length of stay for patients with and without diabetes Fig. 3. Time-series plot illustrating trends in the length of stay of lower-extremity amputations among patients with and without diabetes between 2013 and 2023. Separate lines depict minor, major, and overall amputations, stratified by diabetes status. All amputations recorded during the study period are included, i.e. both first and repeat amputations; the unit of analysis is the amputation-related hospitalisation.

58.7% of patients were discharged home, 16.5% to rehabilitation, and 13.7% to nursing facilities. The overall in-hospital mortality was 4.3% (95% CI 4.1–4.5), with the lowest mortality observed in patients with type 1 DM (1.4% [95% CI: 0.8 – 2.4]) compared to those with type 2 DM (4.1% [95% CI: 3.8 – 4.4]) and patients without DM (4.8% [95% CI: 4.4 – 5.2]). Similarly, the in-hospital case fatality rate was lowest in patients with type 1 DM. The median length of stay (LOS) was shorter for patients with type 1 DM (14 days, Q1-Q3: 8–27) and non-diabetic patients (14 days, Q1-Q3: 7–26) compared to those with type 2 DM (16 days, Q1-Q3: 9–28). Furthermore, the grade of amputation affected the LOS, with major amputations resulting in prolonged hospitalisation (Table 4, Fig. 3). As shown in Figures S 3.1 and S 3.2, the age distribution of patients undergoing amputation differed by DM status, with patients with type 1 DM undergoing amputation at younger ages. The in-hospital outcomes stratified by amputation level can be depicted from Table S10 and S11.

**Table 4:** In-hospital outcomes and post-discharge destination stratified by diabetes status.

Characteristic	Overall, n = 30,047	No diabetes, n = 11,951	DM1, n = 923	DM2, n = 17,173
Post-discharge destination, n (%)				
Home	17,628 (58.7%)	6687 (56.0%)	658 (71.3%)	10,283 (59.9%)
Nursing home	4104 (13.7%)	1810 (15.1%)	57 (6.2%)	2237 (13.0%)
Rehabilitation clinic	4957 (16.5%)	2028 (17.0%)	148 (16.0%)	2781 (16.2%)
Others	3358 (11.1%)	1426 (11.9%)	60 (6.5%)	1872 (10.9%)
Number of deaths, n (%)	1287 (4.3%)	572 (4.8%)	13 (1.4%)	702 (4.1%)
In-hospital case fatality, rate (95% CI)	4.3 (4.1; 4.5)	4.8 (4.4; 5.2)	1.4 (0.8; 2.4)	4.1 (3.8; 4.4)
Length of stay, median (IQR: Q1-Q3)	15 (8, 27)	14 (7, 26)	14 (8, 27)	16 (9, 28)

Abbreviations: n: number; DM1: Diabetes Mellitus type 1; DM2: Diabetes Mellitus type 2; CI: Confidence Interval; IQR: Interquartile Range (Q1-3).

*Table 4. All amputations recorded during the study period are included, i.e. both first and repeat amputations; the unit of analysis is the amputation-related hospitalisation.

**Fig. 4:**
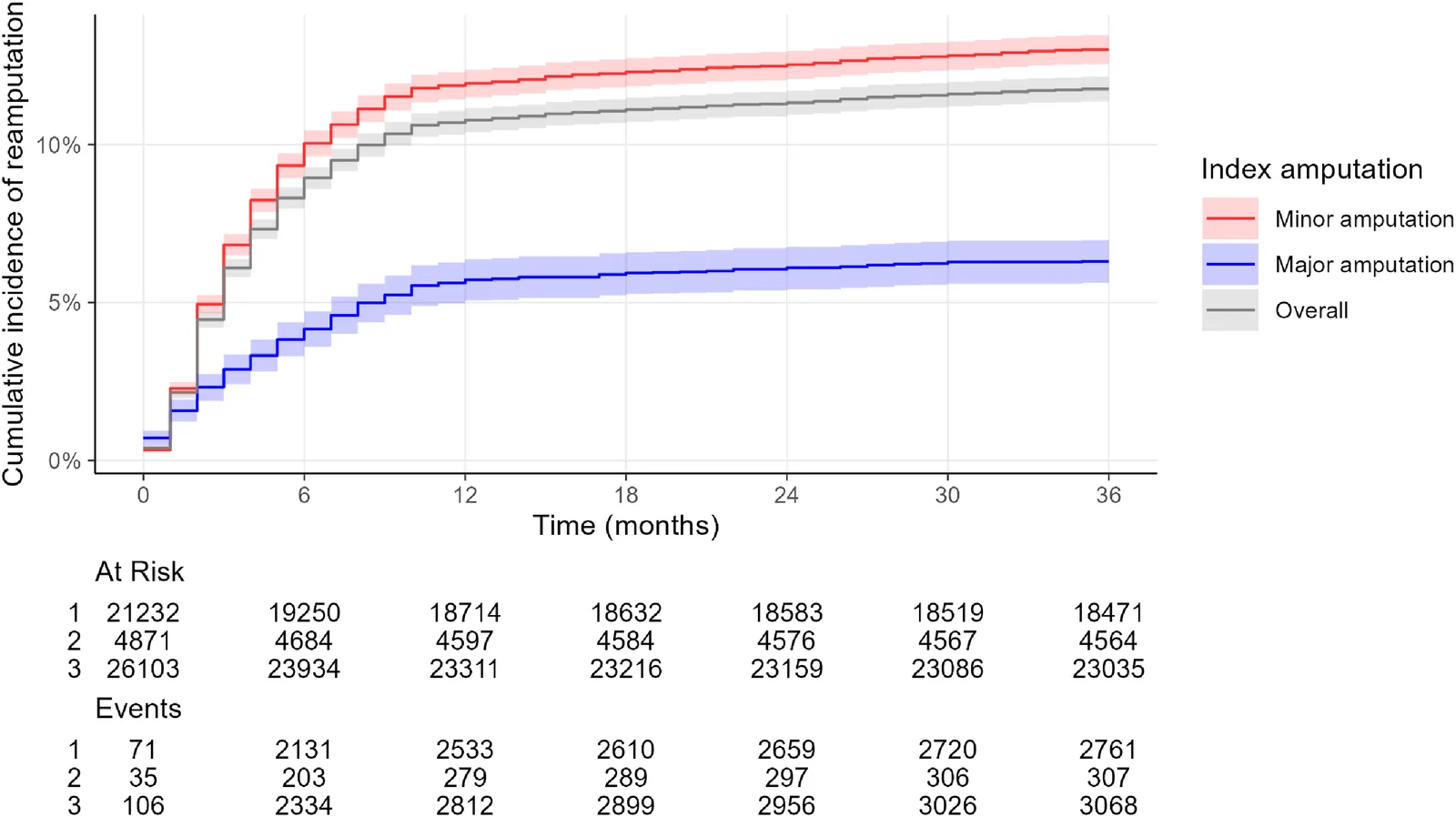
Cumulative incidence of reamputation measured from discharge Fig. 4. Kaplan–Meier-type plot showing the cumulative incidence of re-amputation within 36 months after discharge, stratified by index amputation type (minor, major, and overall). The unit of analysis is the patient: only the first (index) amputation defines study entry and the stratum shown, and any subsequent amputation is counted as an outcome event.

Among patients that survived the initial hospitalisation, re-amputation occurred in 10.0% (95% CI: 9.6–10.4) of patients within six months and increased to 13.0% (95% CI 12.6–13.5) within 36 months (Table S7, Fig. 4). Re-amputation was more frequent after minor amputations (11.8% [95% CI 11.4–12.2]) compared to major amputations (6.3% [95% CI 5.7–7.0]) at 36 months.

## 4 Discussion

Our study presents the first nationwide assessment of LEA trends in Switzerland over the past decade focusing on patients with diabetes. Despite a mild increase in absolute case numbers, age-standardized amputation rates remained stable between 2013 and 2023. Individuals with DM, particularly those with type 2 DM, continued to face a higher risk of amputation compared to individuals without DM, with marked differences in age, sex distribution, and amputation type.

First, while the absolute number of LEAs slightly increased over the decade, mainly driven by minor amputations, the age-standardized amputation rates remained stable in the general population, showing demographic aging as a major influence. These results are consistent with recent multinational analyses, which have reported declining or stable major amputation rates and increasing or stable minor amputation rates in high-income countries [1,9,17,18].

Patients with DM, particularly type 1, continue to experience a higher risk of LEA compared to those without DM, with adjusted incidence rate ratios exceeding 1.5 for any amputation and more than eightfold higher risk among hospitalized patients. Indeed, DM remains the most important risk factor for LEA worldwide and large-scale population-based studies as well as meta-analyses consistently report relative risks ranging from 5- to over 20-fold depending on population and methodology: in England, the relative risk of LEA for individuals with DM compared to those without was over 20 throughout the 2000s, and similar magnitudes have been reported in Germany, Belgium, and other high-income countries [[19], [20], [21]]. The observed decline in the age-standardized amputation proportion among hospitalized patients with DM in our study, particularly the reduction in minor amputations among those with type 2 DM, alongside the general increase in hospitalizations for DM-related foot disease seen in several countries [1,22,23], including Switzerland, has largely been attributed to improvements in preventive care, multidisciplinary management, and greater access to podiatric and vascular services, which has been demonstrated in French and Canadian nationwide studies [24,25].

In our study male sex was a strong independent risk factor for amputation, due to a combination of biological, behavioral, and disease severity factors [[26], [27], [28]]. Epidemiological analyses consistently show that men are more likely to present with deeper, more severe diabetic foot ulcers, higher rates of infection, and a greater prevalence of peripheral arterial disease and neuropathy, all of which are strong independent risk factors of amputation [29,30]. Behavioral risk factors such as higher rates of smoking and delayed healthcare-seeking further contribute to increased risk in men [30,31]. During the study period, the risk of amputation remained stable over the years, with a decrease in amputation rates during the years 2020 and 2021, likely reflecting the impact of the COVID-19 pandemic on healthcare access, with deferred elective procedures, reduced hospital admissions, and disruptions in routine DM and foot care [1,25,32]. However, these temporary declines may mask underlying increases in disease severity and delayed presentations, which could result in future surges in major amputations.

These trends must be interpreted in the context of a changing hospitalized population over the study decade. The progressive shift of lower-acuity care from the inpatient to the ambulatory setting in Switzerland means that the denominator of hospital admissions has itself evolved over time, which may partly explain the declining proportion of admissions accounted for by amputation independently of any true change in amputation risk; rates expressed per general population, which are less sensitive to this shift, remained stable and may therefore provide a more robust picture of the underlying trend. In parallel, surgical decision-making is likely to have changed over a ten-year period, with wider availability of endovascular revascularisation, multidisciplinary foot services and a tendency towards more distal, limb-preserving procedures, which may have contributed to the relative increase in minor amputations alongside stable major amputation rates. The administrative data does not allow these mechanisms to be disentangled, and the observed trends should be read as the net result of changing population structure, care pathways and surgical practice rather than of any single factor.

We found that patients without DM have a greater in-hospital case fatality after lower extremity amputation compared to those with DM. This counterintuitive finding might primarily be explained by differences in patient age, amputation level, and the acuity of presentation. Previous data show that patients without DM undergoing amputation are typically older and more likely to present with advanced limb ischaemia, often requiring more proximal (above-knee) amputations, which are associated with higher perioperative mortality. In contrast, patients with DM tend to undergo amputation at a younger age and at lower anatomical levels, such as minor or below-knee amputations, which carry lower short-term mortality risk. Importantly, peripheral arterial disease and limb ischaemia are not confined to non-diabetic patients: diabetes is itself a major risk factor for ischaemia, and in our cohort PAD was in fact more prevalent among patients with type 2 diabetes (72.5%) than among those without diabetes (53.3%, Table 1). In the non-diabetic group, LEA is driven predominantly by atherosclerotic peripheral arterial disease and chronic limb-threatening ischaemia in older patients with a high cardiovascular comorbidity burden, as well as by acute limb ischaemia and severe soft-tissue or bone infection; these presentations more often require urgent and more proximal amputation, which carries higher perioperative mortality. Age is the strongest independent predictor of early mortality after amputation, with the risk increasing sharply in those over 75 years. Long-term mortality after amputation is higher in patients with DM, with five-year mortality rates exceeding 50% in patients with DM and 60% following a major amputation, reflecting the chronic nature of their disease and cumulative comorbidity burden [[33], [34], [35], [36], [37], [38], [39]].

LOS is a critical outcome after LEA, reflecting both perioperative complexity and patient recovery. In our cohort, median LOS was shorter for patients with type 1 DM and patients without diabetes, which may be due to younger age and fewer comorbidities. However, overall LOS was longer in patients with type 2 DM, after major amputation, as also shown in other cohorts. A Canadian study [40] found median LOS of 11 days for major LEA and 5 days for minor LEA, with diabetes independently associated with prolonged LOS. Danish registry data [41] similarly report median LOS of 13–19 days after major LEA, with a trend toward shorter stays over time but persistent high readmission rates. In diabetic foot ulcer populations, major amputation is associated with the longest hospital stays, and comorbidities such as sepsis, stroke, and chronic kidney disease further increase LOS [[42], [43], [44], [45]].

Post-discharge destination provides a complementary marker of the functional burden and care needs after LEA. In our cohort, most patients were discharged home (58.7%), but a substantial minority required rehabilitation (16.5%) or nursing-home care (13.7%), reflecting the loss of independence and need for post-acute support that frequently follow amputation. These needs differed by diabetes status: discharge home was most frequent among the younger patients with type 1 diabetes (71.3%), whereas discharge to a nursing home was most frequent among patients without diabetes (15.1%), in keeping with their older age. Together with the prolonged length of stay after major amputation, these findings underline that the in-hospital episode captures only part of the total care burden, much of which is borne in rehabilitation and long-term care settings.

Our cohort presented with a higher risk of re-amputation, especially among patients that underwent a minor amputation. This might be due to the persistence of underlying pathologies such as infection, poor vascular supply, and neuropathy, which are often not fully addressed by the initial minor procedure, frequently leaving residual tissue that remains vulnerable to ongoing ischaemia and recurrent ulceration, leading to delayed wound healing and progression of local disease. The risk of re-amputation is highest in the first year after the initial minor amputation, with up to 25% of patients requiring further limb amputation within five years [43,[46], [47], [48]].

Finally, LEA carries a substantial economic and societal burden, including both direct medical costs and indirect costs from disability and productivity loss. Direct expenditures encompass hospitalisation, surgery, rehabilitation, prosthetics, and long-term complication management. While Swiss data are lacking, Belgian data report median costs for major LEA in patients with diabetes exceeding €49,000 in the year before and €45,000 in the year after amputation, with higher costs in those requiring multiple procedures [49]. In the United States, inpatient care for patients with diabetic foot ulcers alone exceeded $790 million in 2010. The average inpatient costs are estimated at $54,000 per patient, and annual post-amputation care costs exceed $55,700 per patient-year, mainly driven by prolonged hospitalizations, readmissions, and ongoing rehabilitation [[50], [51], [52]]. Indirect costs are similarly high: over 50% of patients become unemployed after major amputation [53], resulting in an average loss of over 1700 productive hours per year [54]. High readmission rates and prolonged hospital stays, averaging 71 hospital days per year up to three years post-amputation, and increased need for social services and home care further amplify the disability burden [53,55,56].

### 4.1 Limitations

This study has several limitations. First, it is based on administrative hospital data, which lack clinical granularity and may be subject to misclassification. Diagnoses and procedures were identified using ICD-10-GM and CHOP codes, which, although standardized and routinely audited in Switzerland, can vary in accuracy and interpretation over time. Amputations itselves were identified using CHOP procedure codes, the classification used to record surgical interventions in Switzerland, rather than ICD-10-GM diagnosis codes, which do not capture the amputation procedure itself. Formal validation for the coding of LEA has not been established, and changes in code definitions across years may have influenced observed trends. As outpatient data and individual clinical parameters were unavailable, underreporting and residual confounding cannot be excluded. Mortality could be assessed only during the index hospitalisation, as the dataset captures in-hospital deaths and is not linked to the national mortality registry; out-of-hospital and longer-term deaths are therefore not recorded. Consequently, out-of-hospital mortality could not be adjusted for in any of the analyses. In particular, deaths occurring after discharge could not be accounted for as a competing risk in the re-amputation analysis: as such deaths are not captured in the dataset, the patients concerned could not be distinguished from event-free survivors and remained in the risk set as apparently event-free, which is likely to have diluted the estimated re-amputation hazard and may therefore have led to an underestimation of the cumulative incidence of re-amputation reported here. For the same reason, the case fatality presented reflects in-hospital deaths only and underestimates total post-amputation mortality. Similarly, because rehabilitation and nursing-home stays are not coded within the index hospitalisation, the in-hospital length of stay reported here may underestimate the total duration of inpatient and post-acute care. We standardized rates to the 2013 European standard population to enable comparison with other European analyses; because the age composition of patients undergoing LEA may itself have changed over the study period, this approach emphasises population-level trends but may obscure changes driven by an evolving case-mix, and standardization to the age structure of the 2013 hospitalized population could yield somewhat different estimates. Finally, because diabetes status was ascertained from the same hospitalisation as the amputation, undiagnosed or uncoded diabetes may have led to misclassification of some comparator hospitalisations, and the diabetes–amputation association should be interpreted as an association at the level of the hospitalisation rather than as a causal estimate.

## 5 Conclusions

In this first nationwide analysis of LEA in Switzerland over the past decade, age-standardized amputation rates remained stable between 2013 and 2023, while the absolute number of procedures rose in parallel with population ageing, driven mainly by minor amputations. People with diabetes, and particularly those with type 2 diabetes, continued to bear a disproportionate burden, although the proportion of diabetes-related hospitalizations resulting in amputation declined, chiefly through fewer minor amputations in type 2 diabetes. Re-amputation was common, especially after an initial minor procedure, and in-hospital case fatality was paradoxically higher in patients without diabetes, who were older and more often underwent proximal amputation. These descriptive findings, derived from administrative data, identify the patient groups at highest risk and the trends that warrant continued surveillance; they cannot, on their own, establish the effectiveness of any specific preventive strategy. They nonetheless support sustained monitoring of national amputation trends and focused attention on high-risk groups, in particular older patients with peripheral arterial disease and patients with type 2 diabetes, as a basis for future evaluation of prevention and limb-salvage efforts.

## CRediT authorship contribution statement

**Alexander Schuster:** Data curation, Writing – original draft. **Simon Wolf:** Data curation, Formal analysis, Writing – review & editing. **Julia Neuenschwander:** Conceptualization, Writing – review & editing. **Madlaina Schöni:** Methodology, Validation, Writing – review & editing. **Felix W.A. Waibel:** Conceptualization, Supervision, Writing – review & editing. **Christina Sydler:** Writing – review & editing. **Lea Attias Widmer:** Methodology, Writing – review & editing. **Faranak Rostami:** Methodology, Writing – review & editing. **Marc Righini:** Methodology, Supervision, Validation, Writing – review & editing. **Nils Kucher:** Conceptualization, Supervision, Writing – original draft, Writing – review & editing. **Stefano Barco:** Methodology, Supervision, Validation, Writing – review & editing. **Davide Voci:** Conceptualization, Data curation, Formal analysis, Methodology, Writing – original draft, Writing – review & editing.

## Declaration of generative AI and AI assisted technologies in the writing process

We declare that Generative AI and AI assisted technologies were not used.

## Conflicts of interest

DV, AS, SW, FWAW, CS, LAW, MR, JN, MS, SB have nothing to disclose. NK reports institutional research grants from Concept Medical, Bard, Bentley, Boston Scientific, INARI, Sanofi, and Bayer; and personal fees from Concept Medical, Bayer, Boston Scientific, and INARI.
