# Supplementary material for: Lower extremity amputations in Switzerland: Trends in patients with and without diabetes, 2013–2023
Source: Int J Cardiol Cardiovasc Risk Prev. 2026 Aug 12;30:200696. doi: 10.1016/j.ijcrp.2026.200696 (PMC13507734; doi:10.1016/j.ijcrp.2026.200696)
Supplement: Supplementary file 1 [file mmc1.docx]

**Supplementary material**

**Supplementary Tables**

**Table S1. Diagnoses as defined by the International Classification of Diseases, 10th revision with German Modification (ICD-10-GM)**

| Grouping | Disease / Purpose | ICD-10-GM codes |
| --- | --- | --- |
| Diabetes mellitus | Type 1 diabetes mellitus | E10.x |
|  | Type 2 diabetes mellitus | E11.x |
| Exclusions | Traumatic amputations | S88, S98, T05, T13, T14.7 |
|  | Malignant neoplasms of bone and cartilage of limbs | C40.x, C41.x |
|  | Malignant neoplasms of connective and soft tissue | C49.x |
| Other relevant codes | Acquired absence of limb (status post amputation, not used for incidence but useful in sensitivity analyses) | Z89.4–Z89.6 |

**Table S2. Procedures defined according to the Swiss Classification of Surgical Interventions (CHOP)**

| Grouping | Procedure (lower extremity) | CHOP codes* |
| --- | --- | --- |
| ****Minor amputations**** | Amputation of toe(s) | 84.11 |
|  | Amputation through foot | 84.12 |
|  | Ankle disarticulation | 84.13 |
|  | Transmalleolar amputation at the ankle joint | 84.14 |
| ****Major amputations**** | Amputation through tibia and fibula (below knee) | 84.15 |
|  | Knee disarticulation | 84.16 |
|  | Amputation through femur (above knee) | 84.17 |
|  | Hip disarticulation | 84.18 |
|  | Hemipelvectomy | 84.19 |
| ****Unspecified**** | Amputation of lower limb, level unspecified | 84.10 |

*Note: exact code suffixes may vary slightly depending on the CHOP version for the given year (2013–2023)

**Table S3. Amputation rate (AR) in the general population (rate per 10,000 general population).**

|  | **Total** | | | **Minor** | | | **Major** | | |
| --- | --- | --- | --- | --- | --- | --- | --- | --- | --- |
|  | **Number** | **AR (95% CI)** | **Age-standardized AR (95% CI)** | **Number** | **AR (95% CI)** | **Age-standardized AR (95% CI)** | **Number** | **AR (95% CI)** | **Age-standardized AR (95% CI)** |
| **Overall** | 30,047 | 3.2 (3.2; 3.3) | 2.0 (2.0; 2.0) | 24,353 | 2.6 (2.6; 2.6) | 1.6 (1.6; 1.6) | 5,694 | 0.6 (0.6; 0.6) | 0.4 (0.4; 0.4) |
| **2013** | 2,447 | 3.0 (2.9; 3.1) | 2.0 (1.9; 2.1) | 1,918 | 2.4 (2.3; 2.5) | 1.6 (1.5; 1.6) | 529 | 0.7 (0.6; 0.7) | 0.4 (0.4; 0.4) |
| **2014** | 2,478 | 3.0 (2.9; 3.1) | 2.0 (1.9; 2.1) | 1,990 | 2.4 (2.3; 2.5) | 1.6 (1.5; 1.6) | 488 | 0.6 (0.5; 0.7) | 0.4 (0.4; 0.5) |
| **2015** | 2,606 | 3.1 (3.0; 3.3) | 2.0 (1.9; 2.1) | 2,057 | 2.5 (2.4; 2.6) | 1.6 (1.5; 1.6) | 549 | 0.7 (0.6; 0.7) | 0.4 (0.4; 0.4) |
| **2016** | 2,686 | 3.2 (3.1; 3.3) | 2.0 (1.9; 2.1) | 2,186 | 2.6 (2.5; 2.7) | 1.6 (1.5; 1.7) | 500 | 0.6 (0.5; 0.7) | 0.4 (0.4; 0.5) |
| **2017** | 2,792 | 3.3 (3.2; 3.4) | 2.1 (2.0; 2.2) | 2,240 | 2.7 (2.5; 2.8) | 1.6 (1.6; 1.7) | 552 | 0.7 (0.6; 0.7) | 0.4 (0.4; 0.4) |
| **2018** | 2,730 | 3.2 (3.1; 3.3) | 2.0 (1.9; 2.1) | 2,217 | 2.6 (2.5; 2.7) | 1.6 (1.5; 1.7) | 513 | 0.6 (0.6; 0.7) | 0.4 (0.4; 0.5) |
| **2019** | 2,907 | 3.4 (3.3; 3.5) | 2.1 (2.0; 2.1) | 2,411 | 2.8 (2.7; 2.9) | 1.7 (1.6; 1.8) | 496 | 0.6 (0.5; 0.6) | 0.4 (0.4; 0.4) |
| **2020** | 2,549 | 3.0 (2.8; 3.1) | 1.8 (1.7; 1.9) | 2,080 | 2.4 (2.3; 2.5) | 1.5 (1.4; 1.5) | 469 | 0.5 (0.5; 0.6) | 0.4 (0.3; 0.4) |
| **2021** | 2,744 | 3.2 (3.0; 3.3) | 1.9 (1.8; 2.0) | 2,249 | 2.6 (2.5; 2.7) | 1.5 (1.5; 1.6) | 495 | 0.6 (0.5; 0.6) | 0.4 (0.3; 0.4) |
| **2022** | 2,965 | 3.4 (3.3; 3.5) | 2.0 (1.9; 2.1) | 2,411 | 2.7 (2.6; 2.9) | 1.6 (1.5; 1.7) | 554 | 0.6 (0.6; 0.7) | 0.4 (0.3; 0.4) |
| **2023** | 3,143 | 3.5 (3.4; 3.7) | 2.1 (2.0; 2.1) | 2,594 | 2.9 (2.8; 3.0) | 1.7 (1.6; 1.8) | 549 | 0.6 (0.6; 0.7) | 0.4 (0.4; 0.4) |

Abbreviations: **AR: Amputation Rate**; **CI: Confidence Interval**

*Table S3. All amputations recorded during the study period are included, i.e. both first and repeat amputations; the unit of analysis is the amputation-related hospitalisation.

**Table S4. Amputation rate in people with diabetes (rate per 10,000 general population) and without diabetes (rate per 10,000 general population).**

|  |  |  | **Overall** | **2013** | **2014** | **2015** | **2016** | **2017** | **2018** | **2019** | **2020** | **2021** | **2022** | **2023** |
| --- | --- | --- | --- | --- | --- | --- | --- | --- | --- | --- | --- | --- | --- | --- |
| **Patients with diabetes mellitus** | **Total** | **Number** | 18,096 | 1,364 | 1,407 | 1,505 | 1,594 | 1,668 | 1,686 | 1,798 | 1,550 | 1,719 | 1,838 | 1,967 |
|  |  | **AR (95% CI)** | 1.9 (1.9; 2.0) | 1.7 (1.6; 1.8) | 1.7 (1.6; 1.8) | 1.8 (1.7; 1.9) | 1.9 (1.8; 2.0) | 2.0 (1.9; 2.1) | 2.0 (1.9; 2.1) | 2.1 (2.0; 2.2) | 1.8 (1.7; 1.9) | 2.0 (1.9; 2.1) | 2.1 (2.0; 2.2) | 2.2 (2.1; 2.3) |
|  |  | **Age-standardized AR (95% CI)** | 1.2 (1.2; 1.2) | 1.1 (1.1; 1.2) | 1.1 (1.1; 1.2) | 1.2 (1.1; 1.2) | 1.2 (1.2; 1.3) | 1.3 (1.2; 1.3) | 1.3 (1.2; 1.3) | 1.3 (1.2; 1.4) | 1.1 (1.1; 1.2) | 1.2 (1.1; 1.3) | 1.3 (1.2; 1.3) | 1.3 (1.3; 1.4) |
|  | **Minor** | **Number** | 15,028 | 1,117 | 1,182 | 1,207 | 1,311 | 1,366 | 1,417 | 1,537 | 1,262 | 1,420 | 1,539 | 1,670 |
|  |  | **AR (95% CI)** | 1.6 (1.6; 1.6) | 1.4 (1.3; 1.5) | 1.4 (1.4; 1.5) | 1.5 (1.4; 1.5) | 1.6 (1.5; 1.7) | 1.6 (1.5; 1.7) | 1.7 (1.6; 1.8) | 1.8 (1.7; 1.9) | 1.5 (1.4; 1.5) | 1.6 (1.5; 1.7) | 1.8 (1.7; 1.8) | 1.9 (1.8; 2.0) |
|  |  | **Age-standardized AR (95% CI)** | 1.0 (1.0; 1.0) | 0.9 (0.9; 1.0) | 1.0 (0.9; 1.0) | 0.9 (0.9; 1.0) | 1.0 (0.9; 1.1) | 1.0 (1.0; 1.1) | 1.1 (1.0; 1.1) | 1.1 (1.1; 1.2) | 0.9 (0.9; 1.0) | 1.0 (0.9; 1.0) | 1.0 (1.0; 1.1) | 1.1 (1.1; 1.2) |
|  | **Major** | **Number** | 3,068 | 247 | 225 | 298 | 283 | 302 | 269 | 261 | 288 | 299 | 299 | 297 |
|  |  | **AR (95% CI)** | 0.3 (0.3; 0.3) | 0.3 (0.3; 0.3) | 0.3 (0.2; 0.3) | 0.4 (0.3; 0.4) | 0.3 (0.3; 0.4) | 0.4 (0.3; 0.4) | 0.3 (0.3; 0.4) | 0.3 (0.3; 0.3) | 0.3 (0.3; 0.4) | 0.3 (0.3; 0.4) | 0.3 (0.3; 0.4) | 0.3 (0.3; 0.4) |
|  |  | **Age-standardized AR (95% CI)** | 0.2 (0.2; 0.2) | 0.2 (0.2; 0.2) | 0.2 (0.2; 0.2) | 0.2 (0.2; 0.3) | 0.2 (0.2; 0.3) | 0.2 (0.2; 0.3) | 0.2 (0.2; 0.2) | 0.2 (0.2; 0.2) | 0.2 (0.2; 0.2) | 0.2 (0.2; 0.2) | 0.2 (0.2; 0.2) | 0.2 (0.2; 0.2) |
| **Patients without diabetes mellitus** | **Total** | **Number** | 11,951 | 1,083 | 1,071 | 1,101 | 1,092 | 1,124 | 1,044 | 1,109 | 999 | 1,025 | 1,127 | 1,176 |
|  |  | **AR (95% CI)** | 1.3 (1.3; 1.3) | 1.3 (1.3; 1.4) | 1.3 (1.2; 1.4) | 1.3 (1.3; 1.4) | 1.3 (1.2; 1.4) | 1.3 (1.3; 1.4) | 1.2 (1.2; 1.3) | 1.3 (1.2; 1.4) | 1.2 (1.1; 1.2) | 1.2 (1.1; 1.3) | 1.3 (1.2; 1.4) | 1.3 (1.2; 1.4) |
|  |  | **Age-standardized AR (95% CI)** | 0.8 (0.8; 0.8) | 0.8 (0.8; 0.9) | 0.8 (0.8; 0.9) | 0.8 (0.8; 0.9) | 0.8 (0.7; 0.8) | 0.8 (0.8; 0.9) | 0.7 (0.7; 0.8) | 0.7 (0.7; 0.8) | 0.7 (0.7; 0.7) | 0.7 (0.6; 0.7) | 0.7 (0.7; 0.8) | 0.7 (0.7; 0.8) |
|  | **Minor** | **Number** | 9,325 | 801 | 808 | 850 | 875 | 874 | 800 | 874 | 818 | 829 | 872 | 924 |
|  |  | **AR (95% CI)** | 1.0 (1.0; 1.0) | 1.0 (0.9; 1.1) | 1.0 (0.9; 1.1) | 1.0 (1.0; 1.1) | 1.0 (1.0; 1.1) | 1.0 (1.0; 1.1) | 0.9 (0.9; 1.0) | 1.0 (1.0; 1.1) | 0.9 (0.9; 1.0) | 1.0 (0.9; 1.0) | 1.0 (0.9; 1.1) | 1.0 (1.0; 1.1) |
|  |  | **Age-standardized AR (95% CI)** | 0.6 (0.6; 0.6) | 0.6 (0.6; 0.7) | 0.6 (0.6; 0.7) | 0.6 (0.6; 0.7) | 0.6 (0.6; 0.7) | 0.6 (0.6; 0.7) | 0.5 (0.5; 0.6) | 0.6 (0.5; 0.6) | 0.6 (0.5; 0.6) | 0.5 (0.5; 0.6) | 0.5 (0.5; 0.6) | 0.6 (0.5; 0.6) |
|  | **Major** | **Number** | 2,626 | 282 | 263 | 251 | 217 | 250 | 244 | 235 | 181 | 196 | 255 | 252 |
|  |  | **AR (95% CI)** | 0.3 (0.3; 0.3) | 0.3 (0.3; 0.4) | 0.3 (0.3; 0.4) | 0.3 (0.3; 0.3) | 0.3 (0.2; 0.3) | 0.3 (0.3; 0.3) | 0.3 (0.3; 0.3) | 0.3 (0.2; 0.3) | 0.2 (0.2; 0.2) | 0.2 (0.2; 0.3) | 0.3 (0.3; 0.3) | 0.3 (0.2; 0.3) |
|  |  | **Age-standardized AR (95% CI)** | 0.2 (0.2; 0.2) | 0.2 (0.2; 0.3) | 0.2 (0.2; 0.3) | 0.2 (0.2; 0.2) | 0.2 (0.1; 0.2) | 0.2 (0.2; 0.2) | 0.2 (0.2; 0.2) | 0.2 (0.2; 0.2) | 0.1 (0.1; 0.2) | 0.1 (0.1; 0.2) | 0.2 (0.2; 0.2) | 0.2 (0.2; 0.2) |

Abbreviations: **AR: Amputation Rate**; **CI: Confidence Interval**

*Table S4. All amputations recorded during the study period are included, i.e. both first and repeat amputations; the unit of analysis is the amputation-related hospitalisation.

**Table S5. Amputation proportion (AP) in people with diabetes (rate per 1,000 diabetes-related hospitalizations) and without diabetes (rate per 1,000 non diabetes-related hospitalizations).**

|  |  |  | **Overall** | **2013** | **2014** | **2015** | **2016** | **2017** | **2018** | **2019** | **2020** | **2021** | **2022** | **2023** |
| --- | --- | --- | --- | --- | --- | --- | --- | --- | --- | --- | --- | --- | --- | --- |
| Patients with diabetes mellitus | Total | Number | 18,096 | 1,364 | 1,407 | 1,505 | 1,594 | 1,668 | 1,686 | 1,798 | 1,550 | 1,719 | 1,838 | 1,967 |
|  |  | AP (95% CI) | 12.6 (12.4; 12.8) | 13.4 (12.7; 14.1) | 12.9 (12.3; 13.6) | 12.8 (12.2; 13.5) | 13.1 (12.5; 13.7) | 13.1 (12.5; 13.7) | 12.7 (12.1; 13.3) | 12.8 (12.2; 13.4) | 11.2 (10.6; 11.7) | 11.9 (11.4; 12.5) | 12.2 (11.7; 12.8) | 12.8 (12.3; 13.4) |
|  |  | Age-standardized AP (95% CI) | 5.9 (5.7; 7.4) | 7.3 (6.3; 34.6) | 6.9 (5.7; 24.8) | 6.0 (5.1; 16.5) | 4.8 (4.3; 22.9) | 5.3 (4.7; 14.0) | 6.3 (5.4; 33.7) | 6.1 (5.3; 62.7) | 5.7 (5.0; 8.3) | 5.4 (4.7; 23.4) | 6.1 (5.4; 16.6) | 6.0 (5.4; 24.0) |
|  |  | Hospitalization-standardized AP (95% CU)* | 9.1 (8.9; 16.1) | 10.2 (9.4; 142.7) | 9.9 (9.0; 97.1) | 9.2 (8.5; 60.7) | 8.5 (8; 96.2) | 8.8 (8.3; 51.4) | 9.3 (8.6; 142.1) | 9.2 (8.6; 278.6) | 8.3 (7.7; 9.1) | 8.4 (7.9; 96.2) | 9.1 (8.4; 60.5) | 9.2 (8.6; 96.7) |
|  | Minor | Number | 15,028 | 1,117 | 1,182 | 1,207 | 1,311 | 1,366 | 1,417 | 1,537 | 1,262 | 1,420 | 1,539 | 1,670 |
|  |  | AP (95% CI) | 10.5 (10.3; 10.6) | 11.0 (10.4; 11.6) | 10.9 (10.3; 11.5) | 10.3 (9.7; 10.9) | 10.8 (10.2; 11.4) | 10.7 (10.2; 11.3) | 10.7 (10.1; 11.2) | 10.9 (10.4; 11.5) | 9.1 (8.6; 9.6) | 9.8 (9.3; 10.4) | 10.2 (9.7; 10.8) | 10.9 (10.4; 11.4) |
|  |  | Age-standardized AP (95% CI) | 4.9 (4.7; 6.5) | 6.3 (5.3; 33.8) | 6.1 (5.0; 24.1) | 4.8 (3.9; 15.5) | 3.8 (3.5; 22.2) | 4.3 (3.8; 13.1) | 5.2 (4.5; 32.9) | 5.1 (4.4; 62.1) | 4.4 (3.8; 6.9) | 4.5 (3.9; 22.7) | 5.1 (4.4; 15.7) | 5.2 (4.6; 23.3) |
|  |  | Hospitalization-standardized AP (95% CU)* | 7.5 (7.4; 14.7) | 8.6 (7.8; 141.7) | 8.5 (7.6; 96.2) | 7.4 (6.7; 59.3) | 6.9 (6.5; 95.2) | 7.2 (6.7; 50.2) | 7.8 (7.2; 141.2) | 7.9 (7.3; 277.8) | 6.6 (6.1; 7.4) | 7 (6.5; 95.2) | 7.6 (7; 59.5) | 7.9 (7.3; 95.8) |
|  | Major | Number | 3,068 | 247 | 225 | 298 | 283 | 302 | 269 | 261 | 288 | 299 | 299 | 297 |
|  |  | AP (95% CI) | 2.1 (2.1; 2.2) | 2.4 (2.1; 2.7) | 2.1 (1.8; 2.4) | 2.5 (2.3; 2.8) | 2.3 (2.1; 2.6) | 2.4 (2.1; 2.6) | 2.0 (1.8; 2.3) | 1.9 (1.6; 2.1) | 2.1 (1.9; 2.3) | 2.1 (1.8; 2.3) | 2.0 (1.8; 2.2) | 1.9 (1.7; 2.2) |
|  |  | Age-standardized AP (95% CI) | 1.0 (0.9; 2.6) | 1.0 (0.7; 30.1) | 0.8 (0.6; 20.1) | 1.2 (0.9; 12.5) | 0.9 (0.7; 20.2) | 1.0 (0.7; 10.5) | 1.0 (0.6; 30.1) | 0.9 (0.6; 59.5) | 1.4 (1.0; 4.0) | 0.9 (0.7; 20.2) | 1.0 (0.7; 12.4) | 0.9 (0.6; 20.2) |
|  |  | Hospitalization-standardized AP (95% CU)* | 1.5 (1.4; 9.3) | 1.6 (1.4; 137.6) | 1.4 (1.2; 91.9) | 1.8 (1.5; 55.7) | 1.5 (1.3; 92) | 1.6 (1.3; 46.5) | 1.5 (1.2; 137.5) | 1.4 (1.1; 274.2) | 1.7 (1.4; 2.3) | 1.4 (1.2; 91.9) | 1.5 (1.2; 55.5) | 1.3 (1.2; 91.9) |
| Patients without diabetes mellitus | Total | Number | 11,951 | 1,083 | 1,071 | 1,101 | 1,092 | 1,124 | 1,044 | 1,109 | 999 | 1,025 | 1,127 | 1,176 |
|  |  | AP (95% CI) | 0.8 (0.8; 0.8) | 0.9 (0.8; 0.9) | 0.8 (0.8; 0.9) | 0.8 (0.8; 0.9) | 0.8 (0.8; 0.9) | 0.8 (0.8; 0.9) | 0.8 (0.7; 0.8) | 0.8 (0.8; 0.9) | 0.8 (0.7; 0.8) | 0.8 (0.7; 0.8) | 0.8 (0.8; 0.9) | 0.9 (0.8; 0.9) |
|  |  | Age-standardized AP (95% CI) | 0.4 (0.4; 0.4) | 0.4 (0.4; 0.4) | 0.4 (0.4; 0.5) | 0.4 (0.4; 0.4) | 0.4 (0.3; 0.4) | 0.4 (0.4; 0.4) | 0.4 (0.3; 0.4) | 0.4 (0.3; 0.4) | 0.4 (0.4; 0.4) | 0.4 (0.3; 0.4) | 0.4 (0.3; 0.4) | 0.4 (0.3; 0.4) |
|  |  | Hospitalization-standardized AP (95% CU)* | 0.8 (0.8; 0.8) | 0.9 (0.8; 1.0) | 0.9 (0.8; 0.9) | 0.9 (0.8; 0.9) | 0.8 (0.8; 0.9) | 0.9 (0.8; 0.9) | 0.8 (0.7; 0.8) | 0.8 (0.8; 0.9) | 0.8 (0.7; 0.8) | 0.8 (0.7; 0.8) | 0.8 (0.8; 0.9) | 0.8 (0.8; 0.9) |
|  | Minor | Number | 9,325 | 801 | 808 | 850 | 875 | 874 | 800 | 874 | 818 | 829 | 872 | 924 |
|  |  | AP (95% CI) | 0.6 (0.6; 0.7) | 0.6 (0.6; 0.7) | 0.6 (0.6; 0.7) | 0.6 (0.6; 0.7) | 0.7 (0.6; 0.7) | 0.7 (0.6; 0.7) | 0.6 (0.6; 0.6) | 0.7 (0.6; 0.7) | 0.7 (0.6; 0.7) | 0.6 (0.6; 0.7) | 0.7 (0.6; 0.7) | 0.7 (0.6; 0.7) |
|  |  | Age-standardized AP (95% CI) | 0.3 (0.3; 0.3) | 0.3 (0.2; 0.3) | 0.3 (0.3; 0.3) | 0.3 (0.3; 0.3) | 0.3 (0.2; 0.3) | 0.3 (0.3; 0.3) | 0.2 (0.2; 0.3) | 0.3 (0.2; 0.3) | 0.3 (0.3; 0.3) | 0.3 (0.2; 0.3) | 0.3 (0.2; 0.3) | 0.3 (0.2; 0.3) |
|  |  | Hospitalization-standardized AP (95% CU)* | 0.6 (0.6; 0.7) | 0.7 (0.6; 0.7) | 0.7 (0.6; 0.7) | 0.7 (0.6; 0.7) | 0.7 (0.6; 0.7) | 0.7 (0.6; 0.7) | 0.6 (0.6; 0.6) | 0.6 (0.6; 0.7) | 0.6 (0.6; 0.7) | 0.6 (0.6; 0.7) | 0.6 (0.6; 0.7) | 0.6 (0.6; 0.7) |
|  | Major | Number | 2,626 | 282 | 263 | 251 | 217 | 250 | 244 | 235 | 181 | 196 | 255 | 252 |
|  |  | AP (95% CI) | 0.2 (0.2; 0.2) | 0.2 (0.2; 0.2) | 0.2 (0.2; 0.2) | 0.2 (0.2; 0.2) | 0.2 (0.1; 0.2) | 0.2 (0.2; 0.2) | 0.2 (0.2; 0.2) | 0.2 (0.2; 0.2) | 0.1 (0.1; 0.2) | 0.1 (0.1; 0.2) | 0.2 (0.2; 0.2) | 0.2 (0.2; 0.2) |
|  |  | Age-standardized AP (95% CI) | 0.1 (0.1; 0.1) | 0.1 (0.1; 0.1) | 0.1 (0.1; 0.2) | 0.1 (0.1; 0.1) | 0.1 (0.1; 0.1) | 0.1 (0.1; 0.1) | 0.1 (0.1; 0.1) | 0.1 (0.1; 0.1) | 0.1 (0.1; 0.1) | 0.1 (0.1; 0.1) | 0.1 (0.1; 0.1) | 0.1 (0.1; 0.1) |
|  |  | Hospitalization-standardized AP (95% CU)* | 0.2 (0.2; 0.2) | 0.2 (0.2; 0.3) | 0.2 (0.2; 0.2) | 0.2 (0.2; 0.2) | 0.2 (0.1; 0.2) | 0.2 (0.2; 0.2) | 0.2 (0.2; 0.2) | 0.2 (0.2; 0.2) | 0.1 (0.1; 0.2) | 0.2 (0.1; 0.2) | 0.2 (0.2; 0.2) | 0.2 (0.2; 0.2) |

Abbreviations: **AP: Amputation Proportion**; **CI: Confidence Interval***AP was standardized based on the European standard population 2013 (age-standardized AP) and based on the age distribution of all hospitalizations in Switzerland in 2013 (hospitalization-standardized AP).

*Table S5. All amputations recorded during the study period are included, i.e. both first and repeat amputations; the unit of analysis is the amputation-related hospitalisation.

**Table S6. Amputation proportion (AP) in people with diabetes type 1 (rate per 1,000 diabetes type 1-related hospitalizations) and with diabetes type 2 (rate per 1,000 diabetes type 2-related hospitalizations).**

|  |  |  | **Overall** | **2013** | **2014** | **2015** | **2016** | **2017** | **2018** | **2019** | **2020** | **2021** | **2022** | **2023** |
| --- | --- | --- | --- | --- | --- | --- | --- | --- | --- | --- | --- | --- | --- | --- |
| Patients with diabetes mellitus type 1 | Total | Number | 923 | 65 | 86 | 70 | 72 | 81 | 81 | 86 | 84 | 81 | 105 | 112 |
|  |  | AP (95% CI) | 16.3 (15.3; 17.4) | 14.4 (11.3; 18.3) | 19.5 (15.8; 24.0) | 15.3 (12.1; 19.2) | 14.8 (11.8; 18.6) | 16.6 (13.4; 20.6) | 15.7 (12.7; 19.5) | 15.6 (12.7; 19.3) | 16.3 (13.2; 20.1) | 14.7 (11.8; 18.2) | 18.0 (14.9; 21.7) | 18.5 (15.4; 22.3) |
|  |  | Age-standardized AP (95% CI) | 11.1 (10.3; 12.9) | 10.3 (7.7; 37.4) | 13.6 (10.6; 31.4) | 10.0 (7.4; 20.8) | 8.7 (6.7; 26.5) | 10.6 (8.3; 19.7) | 11.0 (8.5; 37.9) | 11.4 (9.0; 66.7) | 10.9 (8.5; 14.9) | 9.9 (7.6; 36.9) | 13.2 (10.4; 24.0) | 11.9 (9.5; 29.5) |
|  |  | Hospitalization-standardized AP (95% CU)* | 15.9 (14.8; 23.2) | 13.6 (10.3; 145) | 19.6 (15.4; 104.6) | 16.2 (12.4; 66.5) | 15.0 (11.3; 101) | 15.5 (12.1; 57.1) | 15.4 (12.1; 146.2) | 14.5 (11.5; 281.7) | 15.7 (12.4; 19.9) | 13.7 (10.8; 145) | 17.4 (14.2; 67.4) | 18.1 (14.7; 103.3) |
|  | Minor | Number | 759 | 51 | 75 | 61 | 59 | 68 | 68 | 73 | 62 | 64 | 86 | 92 |
|  |  | AP (95% CI) | 13.4 (12.5; 14.4) | 11.3 (8.6; 14.8) | 17.0 (13.6; 21.3) | 13.3 (10.4; 17.0) | 12.2 (9.4; 15.7) | 13.9 (11.0; 17.6) | 13.2 (10.4; 16.7) | 13.3 (10.6; 16.7) | 12.0 (9.4; 15.4) | 11.6 (9.1; 14.8) | 14.7 (11.9; 18.1) | 15.2 (12.4; 18.6) |
|  |  | Age-standardized AP (95% CI) | 9.0 (8.3; 10.8) | 8.0 (5.7; 35.3) | 11.6 (8.9; 29.4) | 8.6 (6.3; 19.4) | 7.1 (5.3; 25.1) | 8.9 (6.7; 17.9) | 9.2 (6.9; 36.3) | 9.8 (7.5; 65.4) | 8.0 (6.0; 11.6) | 7.8 (5.8; 35.1) | 10.4 (8.0; 21.2) | 9.5 (7.4; 27.3) |
|  |  | Hospitalization-standardized AP (95% CU)* | 13.2 (12.2; 20.5) | 10.9 (7.9; 143.2) | 17.6 (13.5; 103) | 14.2 (10.7; 64.8) | 12.5 (9.1; 99.2) | 13.2 (10.1; 55.2) | 13.2 (10.1; 144.7) | 12.4 (9.6; 280.5) | 11.1 (8.4; 14.7) | 10.9 (8.3; 143.2) | 14.4 (11.4; 64.9) | 15.2 (12.1; 101.1) |
|  | Major | Number | 164 | 14 | 11 | 9 | 13 | 13 | 13 | 13 | 22 | 17 | 19 | 20 |
|  |  | AP (95% CI) | 2.9 (2.5; 3.4) | 3.1 (1.8; 5.2) | 2.5 (1.4; 4.5) | 2.0 (1.0; 3.7) | 2.7 (1.6; 4.6) | 2.7 (1.6; 4.5) | 2.5 (1.5; 4.3) | 2.4 (1.4; 4.0) | 4.3 (2.8; 6.4) | 3.1 (1.9; 4.9) | 3.2 (2.1; 5.1) | 3.3 (2.1; 5.1) |
|  |  | Age-standardized AP (95% CI) | 2.1 (1.7; 3.7) | 2.4 (1.2; 31.0) | 2.0 (1.0; 21.0) | 1.4 (0.6; 12.8) | 1.5 (0.8; 20.6) | 1.8 (0.9; 11.1) | 1.8 (0.9; 30.6) | 1.6 (0.8; 59.9) | 2.9 (1.7; 6.1) | 2.1 (1.1; 30.8) | 2.7 (1.5; 13.9) | 2.4 (1.4; 21.2) |
|  |  | Hospitalization-standardized AP (95% CU)* | 2.7 (2.3; 10.5) | 2.7 (1.4; 138.2) | 2 (1.0; 92.3) | 2 (0.9; 55.8) | 2.5 (1.3; 92.5) | 2.3 (1.2; 46.9) | 2.3 (1.2; 138) | 2.1 (1.1; 274.6) | 4.6 (2.8; 7.4) | 2.8 (1.6; 138.3) | 3 (1.8; 56.5) | 2.9 (1.7; 92.8) |
| Patients with diabetes mellitus type 2 | Total | Number | 17,173 | 1,299 | 1,321 | 1,435 | 1,522 | 1,587 | 1,605 | 1,712 | 1,466 | 1,638 | 1,733 | 1,855 |
|  |  | AP (95% CI) | 12.4 (12.3; 12.6) | 13.4 (12.7; 14.1) | 12.7 (12.0; 13.4) | 12.8 (12.1; 13.4) | 13.0 (12.4; 13.7) | 12.9 (12.3; 13.6) | 12.6 (12.0; 13.2) | 12.6 (12.1; 13.2) | 11.0 (10.4; 11.6) | 11.8 (11.2; 12.4) | 12.0 (11.5; 12.6) | 12.6 (12.0; 13.2) |
|  |  | Age-standardized AP (95% CI) | 5.3 (5.1; 62.2) | 7.0 (5.6; 262.0) | 6.1 (4.6; 89.6) | 5.0 (4.4; 260.9) | 4.5 (4.1; 131.6) | 5.2 (4.2; 132.0) | 5.3 (4.7; 89.1) | 5.4 (4.6; 261.1) | 5.4 (4.6; 239.0) | 4.9 (4.3; 260.8) | 5.1 (4.4; 131.9) | 5.4 (4.8; 239.0) |
|  |  | Hospitalization-standardized AP (95% CU)* | 8.5 (8.3; 278.2) | 10.0 (8.9; 56.2) | 9.2 (8.1; 23.8) | 8.4 (7.9; 43.1) | 8.2 (7.7; 30.7) | 8.6 (7.9; 31.1) | 8.6 (8.0; 23.2) | 8.6 (8.0; 43.3) | 8.0 (7.3; 54.6) | 8.0 (7.4; 277.8) | 8.2 (7.6; 28.2) | 8.6 (8.1; 55.1) |
|  | Minor | Number | 14,269 | 1,066 | 1,107 | 1,146 | 1,252 | 1,298 | 1,349 | 1,464 | 1,200 | 1,356 | 1,453 | 1,578 |
|  |  | AP (95% CI) | 10.3 (10.2; 10.5) | 11.0 (10.3; 11.6) | 10.6 (10.0; 11.2) | 10.2 (9.6; 10.8) | 10.7 (10.1; 11.3) | 10.6 (10.0; 11.2) | 10.6 (10.0; 11.1) | 10.8 (10.3; 11.4) | 9.0 (8.5; 9.5) | 9.8 (9.3; 10.3) | 10.1 (9.6; 10.6) | 10.7 (10.2; 11.2) |
|  |  | Age-standardized AP (95% CI) | 4.5 (4.2; 61.6) | 6.3 (4.8; 261.6) | 5.5 (4.0; 89.2) | 3.9 (3.4; 260.3) | 3.6 (3.2; 131.1) | 4.0 (3.5; 131.3) | 4.5 (3.9; 88.6) | 4.5 (3.8; 260.6) | 4.1 (3.5; 238.3) | 4.1 (3.5; 260.4) | 4.4 (3.7; 131.5) | 4.7 (4.1; 238.6) |
|  |  | Hospitalization-standardized AP (95% CU) | 7.1 (6.9; 277.4) | 8.5 (7.5; 55.1) | 7.9 (6.9; 22.7) | 6.7 (6.2; 41.8) | 6.7 (6.3; 29.4) | 6.9 (6.4; 29.6) | 7.2 (6.7; 22) | 7.3 (6.8; 42.2) | 6.3 (5.8; 53.5) | 6.6 (6.1; 277.1) | 7 (6.4; 27.1) | 7.4 (6.9; 54.2) |
|  | Major | Number | 2,904 | 233 | 214 | 289 | 270 | 289 | 256 | 248 | 266 | 282 | 280 | 277 |
|  |  | AP (95% CI) | 2.1 (2.0; 2.2) | 2.4 (2.1; 2.7) | 2.1 (1.8; 2.3) | 2.6 (2.3; 2.9) | 2.3 (2.0; 2.6) | 2.4 (2.1; 2.6) | 2.0 (1.8; 2.3) | 1.8 (1.6; 2.1) | 2.0 (1.8; 2.2) | 2.0 (1.8; 2.3) | 1.9 (1.7; 2.2) | 1.9 (1.7; 2.1) |
|  |  | Age-standardized AP (95% CI) | 0.9 (0.8; 59.5) | 0.7 (0.6; 258.6) | 0.6 (0.5; 86.4) | 1.1 (0.8; 258.8) | 0.9 (0.7; 129.6) | 1.2 (0.5; 129.7) | 0.8 (0.6; 86.5) | 0.9 (0.5; 258.7) | 1.3 (0.9; 236.8) | 0.8 (0.6; 258.6) | 0.7 (0.6; 129.5) | 0.7 (0.5; 236.5) |
|  |  | Hospitalization-standardized AP (95% CU)* | 1.4 (1.3; 274.3) | 1.4 (1.2; 50.3) | 1.2 (1.1; 17.2) | 1.7 (1.5; 38.4) | 1.5 (1.3; 25.6) | 1.7 (1.2; 25.8) | 1.3 (1.1; 17.3) | 1.3 (1; 38.2) | 1.6 (1.3; 50.5) | 1.3 (1.1; 274.2) | 1.3 (1.1; 22.8) | 1.2 (1.0; 50.2) |

Abbreviations: **AP: Amputation Proportion**; **CI: Confidence Interval**

*AP was standardized based on the European standard population 2013 (age-standardized AP) and based on the age distribution of all hospitalizations in Switzerland in 2013 (hospitalization-standardized AP).

*Table S6. All amputations recorded during the study period are included, i.e. both first and repeat amputations; the unit of analysis is the amputation-related hospitalisation.

**Table S7. Cumulative incidence proportion (CIP) of reamputation with corresponding 95% confidence interval (CI) stratified by the initial amputation.**

|  | **Overall** | | **Major amputation** | | **Minor amputation** | |
| --- | --- | --- | --- | --- | --- | --- |
|  | **Events** | **CIP (95% CI)** | **Events** | **CIP (95% CI)** | **Events** | **CIP (95% CI)** |
| **6 months** | 2,131 | 10.0 (9.6, 10.4) | 203 | 4.2 (3.6, 4.8) | 2,334 | 8.9 (8.6, 9.3) |
| **12 months** | 2,533 | 11.9 (11.5, 12.4) | 279 | 5.7 (5.1, 6.4) | 2,812 | 10.8 (10.4, 11.2) |
| **24 months** | 2,659 | 12.5 (12.1, 13.0) | 297 | 6.1 (5.5, 6.8) | 2,956 | 11.3 (10.9, 11.7) |
| **36 months** | 2,761 | 13.0 (12.6, 13.5) | 307 | 6.3 (5.7, 7.0) | 3,068 | 11.8 (11.4, 12.2) |

**Abbreviations: CIP: Cumulative Incidence Proportion**; **CI: Confidence Interval**

*Table S7. The unit of analysis is the patient: only the first (index) amputation defines study entry and the stratum shown, and any subsequent amputation is counted as an outcome event

**Table S8. Incidence rate ratio of lower extremity amputation in people with diabetes, compared with those without diabetes, adjusted for age calendar year: results of the Poisson models.**

| **Characteristic** | **IRR** | **95% CI** | **p-value** |
| --- | --- | --- | --- |
| Year |  |  |  |
| 2013 | Reference | Reference |  |
| 2014 | 0.99 | 0.93, 1.04 | 0.7 |
| 2015 | 1.02 | 0.96, 1.07 | 0.5 |
| 2016 | 1.03 | 0.97, 1.09 | 0.4 |
| 2017 | 1.05 | 0.99, 1.10 | 0.12 |
| 2018 | 1.00 | 0.95, 1.06 | >0.9 |
| 2019 | 1.04 | 0.99, 1.10 | 0.2 |
| 2020 | 0.89 | 0.84, 0.94 | <0.001 |
| 2021 | 0.94 | 0.89, 0.99 | 0.020 |
| 2022 | 0.99 | 0.94, 1.05 | 0.8 |
| 2023 | 1.04 | 0.98, 1.09 | 0.2 |

Abbreviations: **IRR: Incidence Rate Ratio; CI: Confidence Interval**

*Table S8. All amputations recorded during the study period are included, i.e. both first and repeat amputations; the unit of analysis is the amputation-related hospitalisation.

**Table S9. Incidence rate ratio of the proportion of hospitalization of lower extremity amputation in people with diabetes, compared with those without diabetes, adjusted for calendar year: results of the Poisson models.**

| **Characteristic** | **IRR** | **95% CI** | **p-value** |
| --- | --- | --- | --- |
| Year |  |  |  |
| 2013 | Reference | Reference |  |
| 2014 | 0.96 | 0.91, 1.02 | 0.2 |
| 2015 | 0.96 | 0.90, 1.01 | 0.13 |
| 2016 | 0.95 | 0.90, 1.01 | 0.072 |
| 2017 | 0.96 | 0.91, 1.01 | 0.11 |
| 2018 | 0.91 | 0.86, 0.96 | <0.001 |
| 2019 | 0.93 | 0.88, 0.98 | 0.006 |
| 2020 | 0.83 | 0.79, 0.88 | <0.001 |
| 2021 | 0.86 | 0.82, 0.91 | <0.001 |
| 2022 | 0.88 | 0.84, 0.93 | <0.001 |
| 2023 | 0.92 | 0.87, 0.97 | 0.002 |

Abbreviations: **IRR: Incidence Rate Ratio; CI: Confidence Interval**

*Table S9. All amputations recorded during the study period are included, i.e. both first and repeat amputations; the unit of analysis is the amputation-related hospitalisation.

**Table S10. Baseline characteristics and comorbidities stratified by amputation level**

| Characteristic | Overall, n = 30,047 | Minor amputation,  n =24,353 | Major amputation,  n = 5,694 |
| --- | --- | --- | --- |
| Men, n (%) | 21,084 (70.2%) | 17,003 (69.8%) | 4,081 (71.7%) |
| Age (years), median (IQR: Q1-Q3) | 75 (66, 82) | 75 (66, 83) | 73 (64, 81) |
| Comorbidities, n (%) |  |  |  |
| Obesity, n (%) | 1,285 (4.3%) | 1,026 (4.2%) | 259 (4.5%) |
| Class I | 0 | 0 | 0 |
| Class II | 95 (0.3%) | 72 (0.3%) | 23 (0.4%) |
| Class III | 388 (1.3%) | 316 (1.3%) | 72 (1.3%) |
| Peripheral arterial disease | 19,465 (64.8%) | 15,294 (62.8%) | 4,171 (73.3%) |
| Chronic kidney disease | 11,028 (36.7%) | 8,827 (36.2%) | 2,201 (38.7%) |
| Chronic venous disease | 1,190 (4.0%) | 968 (4.0%) | 222 (3.9%) |
| Arterial hypertension | 20,165 (67.1%) | 16,491 (67.7%) | 3,674 (64.5%) |
| Coronary artery disease | 9,568 (31.8%) | 7,540 (31.0%) | 2,028 (35.6%) |
| Heart failure | 4,044 (13.5%) | 2,976 (12.2%) | 1,068 (18.8%) |
| Alcohol abuse | 1,352 (4.5%) | 995 (4.1%) | 357 (6.3%) |
| Foot ulcer | 10,296 (34.3%) | 8,205 (33.7%) | 2,091 (36.7%) |
| Osteomyelitis | 13,568 (45.2%) | 12,058 (49.5%) | 1,510 (26.5%) |
| Sensory neuropathy | 10,639 (35.4%) | 9,058 (37.2%) | 1,581 (27.8%) |
| Previous amputation | 4,973 (16.6%) | 3,861 (15.9%) | 1,112 (19.5%) |
| Liver disease | 900 (3.0%) | 588 (2.4%) | 312 (5.5%) |
| Cerebrovascular disease | 1,448 (4.8%) | 1,084 (4.5%) | 364 (6.4%) |
| Chronic pulmonary disease | 2,593 (8.6%) | 1,960 (8.0%) | 633 (11.1%) |
| Active or history of cancer | 1,547 (5.1%) | 1,156 (4.7%) | 391 (6.9%) |

Abbreviations: n: number; IQR: Interquartile Range (Q1-Q3)

*Table S10. All amputations recorded during the study period are included, i.e. both first and repeat amputations; the unit of analysis is the amputation-related hospitalisation.

**Table S11. In-hospital outcomes and post-discharge destination stratified by amputation level.**

| **Characteristic** | **Overall,**  **n = 30,047** | **Minor amputation,**  **n = 24,353** | **Major amputation,**  **n = 5,694** |
| --- | --- | --- | --- |
| Post-discharge destination, n (%) |  |  |  |
| Home | 17,628 (58.7%) | 16,824 (69.1%) | 804 (14.1%) |
| Nursing home | 4,104 (13.7%) | 2,980 (12.2%) | 1,124 (19.7%) |
| Rehabilitation clinic | 4,957 (16.5%) | 2,561 (10.5%) | 2,396 (42.1%) |
| Others | 3,358 (11.1%) | 1,988 (8.2%) | 1,370 (24.1%) |
| Number of deaths, n (%) | 1,287 (4.3%) | 670 (2.8%) | 617 (10.8%) |
| In-hospital case fatality, rate (95% CI) | 4.3 (4.1; 4.5) | 2.8 (2.6; 3.0) | 10.8 (10.1; 11.7) |
| Length of stay, median (IQR: Q1-Q3) | 15 (8, 27) | 13 (7, 23) | 29 (17, 47) |

Abbreviations: n: number; DM1: Diabetes Mellitus type 1; DM2: Diabetes Mellitus type 2; CI: Confidence Interval; IQR: Interquartile Range (Q1-3);

*Table S11. All amputations recorded during the study period are included, i.e. both first and repeat amputations; the unit of analysis is the amputation-related hospitalisation.

**Supplementary Figures**

**Figure S1. Proportion of hospitalizations (per 1000 hospital admissions) in patients with DM1**

**Figure S1.** Time-series plot illustrating trends in the proportion of hospitalizations (per 1,000 hospital admissions) involving lower extremity amputations among patients with type 1 diabetes mellitus from 2013 to 2023. Separate lines depict minor, major, and overall amputations. All amputations recorded during the study period are included, i.e. both first and repeat amputations; the unit of analysis is the amputation-related hospitalisation.

**Figure S2. Proportion of hospitalizations (per 1000 hospital admissions) in patients with DM2**

**Figure S2**. Time-series plot illustrating trends in the proportion of hospitalizations (per 1,000 hospital admissions) involving lower extremity amputations among patients with type 2 diabetes mellitus from 2013 to 2023. Separate lines depict minor, major, and overall amputations. All amputations recorded during the study period are included, i.e. both first and repeat amputations; the unit of analysis is the amputation-related hospitalisation.

**Figure S 3.1. Absolute number of amputations per age group for patients with DM1**

**Figure S3.1**. Time-series plot illustrating trends in the absolute number of amputations involving lower extremity amputations among patients with type 1 diabetes mellitus from 2013 to 2023. Separate lines depict minor, major, and overall amputations. All amputations recorded during the study period are included, i.e. both first and repeat amputations; the unit of analysis is the amputation-related hospitalisation.

**Figure S 3.2. Absolute number of amputations per age group for patients with DM2**

**Figure S3.2**. Time-series plot illustrating trends in the absolute number of amputations involving lower extremity amputations among patients with type 2 diabetes mellitus from 2013 to 2023. Separate lines depict minor, major, and overall amputations. All amputations recorded during the study period are included, i.e. both first and repeat amputations; the unit of analysis is the amputation-related hospitalisation.
